# A narrative review of the pharmacological management of psychosis in Alzheimer's disease

**DOI:** 10.1192/bjo.2021.462

**Published:** 2021-06-18

**Authors:** Alexander Matthews, Georgina Priest, Sophie Thomas, Hannah White, Stephen De Souza

**Affiliations:** 1 Bristol Medical School; 2 Taunton and Somerset NHS Foundation Trust

## Abstract

**Aims:**

Dementia is estimated to affect 50 million people worldwide, with around 60% of these cases attributable to Alzheimer's disease (AD). One of the common behavioural and psychological symptoms associated with AD is psychosis. Psychosis, experiencing delusions or hallucinations, can be one of the most distressing ordeals for patients with AD, as well as those around them. Effectively managing these symptoms can lead to a vast improvement in life quality. Currently, there are no medications specifically licensed in the UK for the treatment of psychosis in AD. To help guide clinical practice, we reviewed the evidence underpinning the pharmacological treatment of psychosis in AD. The aim of the study was to positively influence clinical practice and thereby improve the life quality of this patient group.

**Method:**

An advanced PubMed search was used to identify studies which investigated the pharmacological treatments for acute psychosis in people with AD. Papers included were double blind, placebo controlled, randomised controlled trials specifically for AD dementia. Papers must have reported their findings using a specific psychosis subscale (PS); examples being “Behavioural Pathology in AD” (BEHAVE-AD-PS), “Brief Psychiatric Rating Scale” (BPRS-PS), and “Neuropsychiatric Inventory - Nursing Home Version” (NPI-NH-PS). Populations of both outpatients and residential patients were accepted. 14 papers, comprising some 3237 patients, were included and critically analysed in the final review.

**Result:**

Risperidone (BEHAVE-AD-PS: -1.3 [p = 0.004] & -1.9 [p = 0.039]; BPRS-PS: -0.5 [p = 0.08]) and aripiprazole (NPI-NH-PS: -1.0 [p = 0.169] & -1.8 [p = 0.013]) successfully reduced psychosis symptoms in patient populations. However, these medications were associated with a statistical increase in severe adverse events including strokes and cognitive decline. Pimavanserin (NPI-NH-PS: -1.9 [p = 0.045]) also offered a notable reduction in psychosis symptoms, but was associated with increased agitation/aggression. Whilst commonly used in clinical practice, quetiapine, olanzapine, and haloperidol showed negligible therapeutic changes compared to placebo using multiple psychosis subscales. Olanzapine and haloperidol were associated with increased rates of severe adverse events including extrapyramidal symptoms. Quetiapine showed limited side effects.

**Conclusion:**

Risperidone and aripiprazole offer effective means to help AD patients cope with psychosis, but these medications also come with an increased risk of developing life-threatening complications. They should, therefore, be administered judiciously. Pimavanserin shows early promise in treating this group of patients, with no life-threatening adverse effects associated with its use. Further research is required before endorsing the use of pimavanserin. There is little evidence to support the therapeutic use of quetiapine, olanzapine, and haloperidol in this patient population. No financial sponsorship declared.

